# Is it enough to utilize a single anchor for repair of rotator cuff tears ≤ 3 * 3 cm²?

**DOI:** 10.1371/journal.pone.0320915

**Published:** 2025-04-21

**Authors:** Ala’ Hawa, Alexander Tham, James Bilbrough, Christyon Hayek, Mina Shenouda, George A. C. Murrell

**Affiliations:** 1 Department of Surgery, Orthopedics Division, Faculty of Medicine, Yarmouk University, Irbid, Jordan; 2 Orthopedic Research Institute, St George Hospital Campus, University of New South Wales, Kogarah, New South Wales, Australia; Shanghai Jiao Tong University Medical School Affiliated Ruijin Hospital, CHINA

## Abstract

**Background:**

Biomechanical studies showed that increasing number of anchors could improve the repair strength of the repaired cuff at time zero.

**Purpose:**

The aim of this study was to determine if utilizing only a single anchor for a cuff tear repair is sufficient or otherwise to give a similar retear rate and clinical outcome as multiple anchors in a matched group of patients.

**Study design:**

Cohort study; Level of evidence, 3.

**Methods:**

Retrospective analysis of 346 matched consecutive patients (single anchor group, n = 173; multiple anchors group, n = 173) who had cuff tears ≤ 3*3 cm² (mediolateral * anteroposterior diameters) repaired by a single senior surgeon. Ultrasound was used to evaluate the integrity of repair 6 months post-surgery. Patient and surgeon reported outcomes were used to evaluate the clinical outcome of the method used for repair.

**Results:**

6 months post-surgery; the retear rate for cuff tears ≤ 1*1 cm², tears ≤ 1 cm in mediolateral diameter and > 1 cm in anteroposterior diameter and tears > 1 cm in mediolateral diameter and ≤ 1 cm in anteroposterior diameter was similar in single and multiple anchors groups (4.8%) (3.3%) (P = 1.00), (10.8%) (7.9%) (P = 0.71) and (0%) (0%) respectively. Retear rate for cuff tears > 1*1 cm² was significantly higher in single anchor group (25.4%) compared to multiple anchors groups (10.9%) (P < 0.05). Operative time was significantly lower in single anchor group (14 minutes) compared to multiple anchors group (20 minutes) (P < 0.05) only for cuff tears ≤ 1*1 cm².

**Conclusion:**

6-months post-surgery; there was no significant difference in retear rate or clinical outcome between patients with tears ≤ 3*3 cm² (mediolateral * anteroposterior diameters) who had their cuff tears repaired using a single anchor compared to those who had their cuff tears repaired using multiple anchors unless both the mediolateral and anteroposterior diameters of the tear were > 1 cm, for which the utilization of multiple anchors showed a significantly lower retear rate at 6 months post-surgery. Operative time was significantly shorter only when a single anchor was used for repair of tears ≤ 1*1 cm².

## Introduction

Rotator cuff tears are considered to be one of the commonest causes of shoulder pain and disability [1,2]. Several methods of fixation have been proposed to provide a strong and secure repair for rotator cuff tears to reduce the pain and disability implicated by them [3–7]. Suture anchors are considered common and effective method of fixation for torn rotator cuffs to their bony footprints [4,8,9].

Several techniques have been proposed, studied and compared to utilize suture anchors in rotator cuff tears repair. Single-row, double-row, and trans osseous equivalent techniques are considered to be the commonest ones used for rotator cuff tears repair [10–13].

Single-row repair is considered to be an effective technique in repairing rotator cuff tears [14–17]. Clinical studies even showed that single-row fixation yields a comparable clinical outcome and rotator cuff integrity to double-row repair for tears with a mediolateral width ≤ 3 cm [18–20].

Biomechanical studies showed that increasing the number of anchors or the complexity of repair method may improve the repair strength at time zero (the time of repair) [5,21,22]. However, to our knowledge, there are no clinical studies comparing the rotator cuff integrity and the clinical outcome after using one versus multiple anchors in single row repair of rotator cuff tears.

Therefore, the aim of this study was to determine if utilizing one anchor in single row rotator cuff repair for small and mediums sized tears ≤ 3 * 3 cm² would give a similar retear rate and clinical outcome in comparison to the usage of multiple anchors in a matched group of patients after 6 months of repair.

## Methods

### Study design

This study was designed to evaluate the outcome of utilizing one anchor in comparison to multiple anchors in single row rotator cuff tears repair using a matched cohort analysis of prospectively collected data. The patients were assigned into two groups; the single anchor group and the multiple anchors group. Both groups were matched using propensity score matching according to age, gender, tear size and shoulder stiffness score (modified L’Insalata Shoulder Rating scale) [23] as those factors are significant predictors for rotator cuff re-tear [19,24–27]. The primary outcome was the repair integrity at 6 months which was evaluated by an experienced independent sonographer (Graduate Dip in Medical Ultrasound). Secondary outcomes were the patients’ ranked pain and function in addition to the examiners’ determined strength and range of motion. The number of anchors used for cuff tears repair was random as the aim was mainly to close the tear gap and cover the footprint of the torn cuff muscles.

### Ethics approval

Ethical approval for this study obtained from the South Eastern Sydney Local Health Network Human Research Ethics Committee–Southern Sector (2019/ETH14049).

This study analyzes data collected blindly on February 1, 2023 for a retrospective patient’s record extending from January 1, 2005 to December 31, 2022. Researchers had access to limited identifying information (e.g., medical record numbers) during data collection for study purposes. All identifying information was removed from the data prior to analysis and publication. Data was stored securely on a password-protected server with limited access

### Inclusion criteria

To be included in this study; patients needed to have had a full thickness rotator cuff tear repaired primarily by a senior surgeon utilizing one or more anchors, have a minimum follow up of 6 months and an ultrasound assessment of repair integrity at 6 months post repair.

### Exclusion criteria

Isolated subscapularis tears, irreparable tears, tears with a mediolateral or anteroposterior diameter > 3 cm, partial-thickness rotator cuff tears, revision surgeries, tears associated with an avulsion fracture, tears repaired with synthetic (PTFE) patches and patients who did not attend the 6 months follow up appointment.

### Surgical technique

All surgeries were performed arthroscopically. Patients received interscalene block and sedation then positioned in the upright beach-chair position. Two incisions were made for repair. A posterolateral incision (2 cm inferior and 1 cm medial to the posterolateral edge of acromion) for the arthroscopic camera and an anterolateral incision for instrumentation (under arthroscopic guidance). Rotator cuffs were repaired in an inverted mattress single row configuration using a suture passer (Opus SmartStitch; Smith & Nephew) and knotless No.2 polyster braided suture anchors (Opus Magnum No. 2; Smith & Nephew) in either undersurface or both surfaces repair techniques [28].

### Postoperative protocol

Patients were discharged on the same day and their operated arm was kept in an abduction pillow (UltraSling; DJO) for 6 weeks post-surgery. Precise instructions were given for the patients including the time and method of exercises needed post-operatively. During the first week after surgery; grip strengthening, elbow flexion and extension and shoulder pendular exercises were recommended. After that, shoulder flexion, extension, internal and external rotation exercises were initiated until the 6 weeks visit post-surgery. Then, active shoulder range of motion and isometric exercises were recommended. Three months after repair; patients were allowed to start lifting objects weighing 5 kgs or more and to do overhead activities. Patients returned for their follow up visits at the 1^st^ week, 6^th^ week and 6 months post-surgery. In addition, ultrasound was performed after 6 months of surgery to evaluate the integrity of the cuff repair [29].

### Datal collection

The data of this study was collected during the period from January 2005 to December 2022.

#### Rotator cuff tear surface area (mm²).

Was the product of both anteroposterior and mediolateral diameters of the tears which were collected intraoperatively using the arthroscopic shaver head diameter as a reference as this method is considered an effective and accurate method for estimation of the cuff tear size arthroscopically [30,31]. In addition, the time of surgery (minutes) was recorded from the beginning of incision until skin closure using a digital clock.

#### Patients reported outcome.

**Fig 1:**
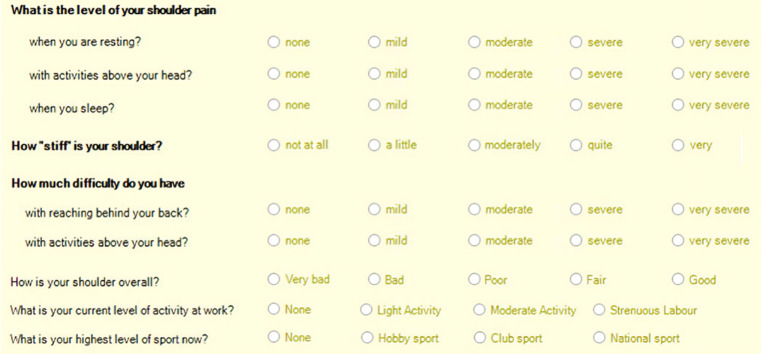
Patients exclusion hierarchy. * Represents a list for patients excluded according to our exclusion criteria. ¶ ML is the mediolateral dimension of the tear, AP is the anteroposterior dimension of the tear & the “faint blue” color coded boxes represent the treatment method used for repair of rotator cuff tears.

The overall patient’s satisfaction about their shoulders, shoulder stiffness, difficulty in movements and level of pain were identified using a self-reported questionnaire (modified L’Insalata Shoulder Rating scale) [23] pre-operatively, at 6 weeks and 6 months post-surgery [Fig 1 - Appendix]. The answers for the questions in the modified questionnaire were converted into parametric values starting from 0 to 4 to yield a numerical score for each one of them.

#### Surgeon reported outcome.

Shoulder passive range of motion (degrees) was measured by an independent orthopedic surgeon using eyeballed physical examination and the strength (newton) of shoulder abduction, adduction, internal rotation and external rotation was quantified using a handheld dynamometer against active movement (HFG 110; Transducer Techniques) pre-operatively, at 6 weeks and 6 months post-surgery [24].

### Statistical analysis

Data in this study are reported as mean ± standard error of the mean (mean ± SEM). Statistical analysis was performed using SPSS version 28.0 (IBM) and statistical significance was set at P < 0.05. Quantitative variables: age, tear size surface area, operative time and patient-rated and surgeon-measured outcomes were compared between groups using independent-samples Mann-Whitney U tests. Categorical variables: gender, site of surgery and retear rate at 6 months were compared between groups using the X² tests. Graphs were generated using GraphPad Prism version 10 (Dotmatics). Propensity score which computes the probability that a unit will enroll in a program based on observed characteristics was used to match the major variables affecting the retear rate as this score is estimated using a logistic regression model, in which treatment status is regressed on observed baseline characteristics [32].

## Results

**Fig 2:**
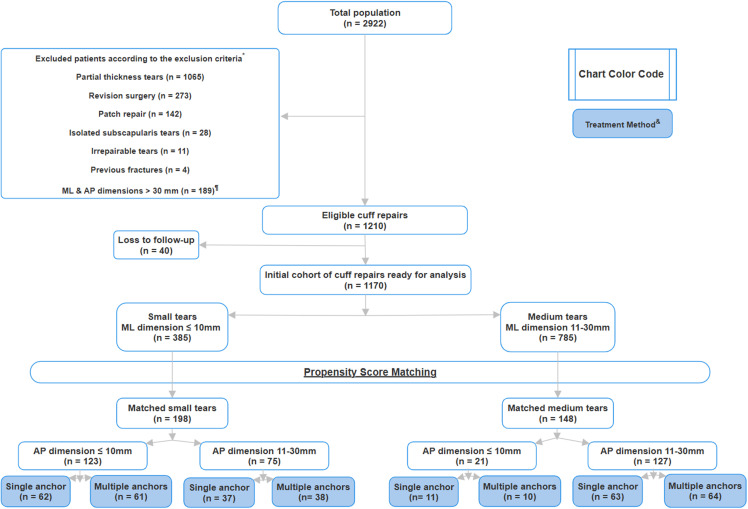
(A & B) Graphical & Tabular representations of retear rates 6 months postoperatively for both single & multiple anchors groups among tear areas of size ML ≤ 10 mm * AP ≤ 10 mm, ML ≤ 10 mm * AP 11-30 mm, ML 11-30 mm * AP ≤ 10 mm and ML 11-30mm * AP 11-30mm. Black bars represent the Single Anchor group, and grey bars represent Multiple Anchors.

During the study period from January 1, 2005 to December 31, 2022, 2922 rotator cuffs were repaired (Fig 2). 1712 patients met the exclusion criteria; 1065 patients had partial thickness tears, 273 patients had revision surgeries, 142 patients were treated with synthetic PTFE patches, 189 patients had tears with mediolateral * anteroposterior diameter > 3 * 3 cm², 28 patients had isolated subscapularis tears, 11 patients had irreparable cuff tears and 4 patients had greater tuberosity fractures leaving 1210 eligible rotator cuff repairs. 40 patients did not attend the 6 months follow up appointment. Therefore, the data for 1170 patients was available for analysis. The data of these patients was arranged into two groups; small (n = 385) and medium (n = 785) size tears groups according to DeOrio and Cofield classification using the cut off value of 1 cm for the mediolateral width of the tear [33]. Then the two groups were matched using propensity score matching according to age, gender, tear size and shoulder stiffness score (modified L’Insalata Shoulder Rating scale) as these factors are significant predictors for rotator cuff re-tear [19,24–27]. In addition, to compare both subjective and objective clinical outcomes for patients pre-operatively, at 6 weeks and 6 months postoperatively; both groups were also matched for patients and surgeon reported outcomes. The matching process included: 1) excluding patients who had tears with mediolateral and anteroposterior diameter > 3 * 3 cm² as those tears inherently require more than an anchor for repair due to the large footprint. Additionally, the senior surgeon had always used multiple anchors for repair of those tears, 2) arranging the cohort of the remaining patients into two groups according to tear size using the mediolateral diameter of the tears as a reference for classification [33], 3) age, gender, tear size and preoperative shoulder stiffness were matched for each group using propensity score matching [Table 1]. Matching based on these variables was done using FUZZY algorithm in SPSS version 28.0 (IBM) with a match tolerance of 0.01 which generated a cohort of 346 patients, 198 patients per small tears group and 148 patients per medium tears group. Then, patients in each group were assigned into 2 subgroups according to the anteroposterior tear diameter; the first subgroup included patients with an anteroposterior tear diameter ≤ 1 cm and the second subgroup included patients with an anteroposterior tear diameter > 1 cm. Each subgroup of patients was separated into two groups; patients treated with a single anchor (n = 173) and patients treated with multiple anchors (n = 173). Gender and tear size were significant (< 0.05) contributors in the generation of the propensity score. Non-significant variables were kept as their inclusion ensured that the highest number of measures were matched [Table 1].

**Table 1:** Propensity score matching.

Matching for Small tears (ML <10mm)			
	**B** *	**Wald** *	**p-value**
Age	0.014	1.271	0.26
Tear Size Anterior Posterior (AP)	−0.329	2.297	0.13
Tear Size Medial Lateral (ML)	−0.185	.479	0.49
Tear Size Area (ML x AP)	0.010	.188	0.67
Gender	−0.541	4.742	**0.03**
Preoperative stiffness	0.007	.006	.94
Constant	3.879	2.214	0.14
Final Number	198		
**Matching for Medium tears (ML 11–30mm)**			
	**B** *	**Wald** *	**p-value**
Age	−0.001	0.006	0.939
Tear Size Anterior Posterior (AP)	−0.506	24.779	**<.001**
Tear Size Medial Lateral (ML)	−0.522	20.206	**<.001**
Tear Size Area (ML x AP)	0.020	15.056	**<.001**
Gender	0.081	.087	0.768
Preoperative stiffness	−0.037	.126	0.722
Constant	9.652	19.351	<.001
Final Number	148		

*Propensity score matching. *B represents the coefficient value for each predictor variable. *Wald is the statistic used to determine significance of each predictor variable, with a higher value indicating a greater contribution to the model.

### Demographics

The summary of demographics for the different groups and subgroups are depicted in [Table 2]. For small tears (mediolateral diameter ≤ 1 cm) with anteroposterior diameter ≤ 1 cm, the single anchor subgroup (n = 62) consisted of 31 males and 31 females with a mean age of 58 years. In the multiple anchors subgroup (n = 61), 33 males and 28 females were included with a mean age of 58 years. In the single anchor subgroup, the ratio of surgeries in right shoulder to left shoulder was 38:24 in comparison to 30:31 in multiple anchors subgroup. Preoperative duration of symptoms was 63 months in the single anchor subgroup in comparison to 63 months in the multiple anchors subgroup. There was no significant difference between the two subgroups in gender ratio, site of surgery ratio, preoperative symptoms duration and the age of patients (P = 0.72) (P = 0.21) (P = 0.39) (P = 0.72) respectively. For small tears (mediolateral diameter ≤ 1 cm) with anteroposterior diameter 1–3 cm, the single anchor subgroup (n = 37) consisted of 16 males and 21 females with a mean age of 62 years. In the multiple anchors subgroup (n = 38), 16 males and 22 females were included with a mean age of 65 years. In the single anchor subgroup, the ratio of surgeries in right shoulder to left shoulder was 21:16 in comparison to 20:18 in multiple anchors subgroup. Preoperative duration of symptoms was 66 months in the single anchor subgroup in comparison to 57 months in the multiple anchors subgroup. There was no significant difference between the two subgroups in gender ratio, site of surgery ratio, preoperative symptoms duration and the age of patients (P = 1.00) (P = 0.82) (P = 0.48) (P = 0.28) respectively. For medium tears (mediolateral diameter 1–3 cm) with anteroposterior diameter ≤ 1 cm, the single anchor subgroup (n = 11) consisted of 5 males and 6 females with a mean age of 62 years. In the multiple anchors subgroup (n = 10), 6 males and 4 females were included with a mean age of 60 years. In the single anchor subgroup, the ratio of surgeries in right shoulder to left shoulder was 4:7 in comparison to 6:4 in multiple anchors subgroup. Preoperative duration of symptoms was 49 months in the single anchor subgroup in comparison to 74 months in the multiple anchors subgroup. There was no significant difference between the two subgroups in gender ratio, site of surgery ratio, preoperative symptoms duration and the age of patients (P = 0.67) (P = 0.40) (P = 0.11) (P = 0.81) respectively. For medium tears (mediolateral diameter 1–3 cm) with anteroposterior diameter > 1–3 cm, the single anchor subgroup (n = 63) consisted of 26 males and 3 females with a mean age of 61 years. In the multiple anchors subgroup (n = 64), 19 males and 45 females were included with a mean age of 60 years. In the single anchor subgroup, the ratio of surgeries in right shoulder to left shoulder was 37:26 in comparison to 33:31 in multiple anchors subgroup. Preoperative duration of symptoms was 72 months in the single anchor subgroup in comparison to 67 months in the multiple anchors subgroup. There was no significant difference between the two subgroups in gender ratio, site of surgery ratio, preoperative symptoms duration and the age of patients (P = 0.20) (P = 0.48) (P = 0.62) (P = 0.51) respectively.

**Table 2:** Patients demographics.

	Small Tears (ML ≤10mm)					
**Tear Size Areas**	**AP ≤10mm**			**AP 11–30mm**		
	Single Anchor	Multiple Anchors	p	Single Anchor	Multiple Anchors	p
**Count**	62	61		37	38	
**Age (years)**	58±1 (15–82)	58±1 (35–84)	0.72	62±2 (36–81)	65±1 (45–82)	0.28
**Sex (male: female)**	31 (50%): 31 (50%)	33 (54%): 28 (46%)	0.72	16 (43%): 21 (57%)	16 (42%): 22 (58%)	1.00
**Shoulder Side (L:R)**	24 (39%): 38 (61%)	31 (51%): 30 (49%)	0.21	16 (43%): 21 (57%)	18 (47%): 20 (53%)	0.82
**Preoperative symptom duration [months]**	63±4 (2–166)	63±8 (3–421)	0.39	66±13 (10–404)	57±2 (49–99)	0.48
**Operative time [min]**	14±1 (3–60)*	20±1 (6–50)*	**0.01**	14±1 (4–30)	17±1 (5–40)	0.19
**Tear size**						
Anterior Posterior [mm]	10±0 (5–10)	10±0 (5–10)	0.45	16±1 (11–30)	16±1 (12–30)	0.99
Medial Lateral [mm]	10±0 (5–10)	10±0 (5–10)	0.69	10±0 (5–10)	10±0 (7–10)	0.64
Area (AP x ML) [mm^2^]	94±2 (25–100)	94±2 (25–100)	0.73	153±6 (75–300)	157±6 (91–300)	0.59
	**Medium Tears (ML 11–30 mm)**					
**Tear Size Areas**	**AP ≤10mm**			**AP 11–30mm**		
	Single Anchor	Multiple Anchors	p	Single Anchor	Multiple Anchors	p
**Count**	11	10		63	64	
**Age (years)**	62±4 (44–87)	60±3 (43–75)	0.81	61±1 (40–81)	60±1 (42–77)	0.51
**Sex (male: female)**	5 (46%): 6 (54%)	6 (60%): 4 (40%)	0.67	26 (41%): 3 (59%)	19 (30%): 45 (70%)	0.20
**Shoulder Side (L:R)**	7 (64%): 4 (36%)	4 (40%): 6 (60%)	0.40	26 (41%): 37 (59%)	31 (48%): 33 (52%)	0.48
**Preoperative symptom duration [months]**	49±21 (3–204)	74±19 (49–183)	0.11	72±6 (9–303)	67±4 (0–146)	0.62
**Operative time [min]**	14±2 (6–30)	16±1 (10–20)	0.39	16±1 (4–50)	19±2 (7–97)	0.40
**Tear size**						
Anterior Posterior [mm]	10±1 (5–10)	10±0 (10–10)	0.76	17±1 (12–30)	17±1 (12–30)	0.44
Medial Lateral [mm]	16±1 (15–20)	16±1 (15–20)	0.71	17±1 (12–30)	16±1 (12–30)	0.07
Area (AP x ML) [mm^2^]	146±5 (100–150)	160±7 (150–200)	0.31	295±20 (144–900)	287±19 (144–900)	0.65

*Represents p <0.05. All data expressed as mean ± SEM with range in brackets.

### Intraoperatively

For small tears (mediolateral diameter ≤ 1 cm) with anteroposterior diameter ≤ 1 cm, in the single anchor subgroup, the average tear length (anteroposterior diameter) and width (mediolateral diameter) were 10 mm and 10 mm respectively. Similarly, the average tear length and width were 10 mm and 10 mm in the multiple anchors subgroup respectively. There was no significant difference in tear diameters (anteroposterior and mediolateral) between both subgroups (P = 0.45) (P = 0.69) respectively. The average operative time for the single anchor subgroup (14 minutes) was significantly lower than the multiple anchors subgroup (20 minutes) (P < 0.01). For small tears (mediolateral diameter ≤ 1 cm) with anteroposterior diameter 1–3 cm, in the single anchor subgroup, the average tear length and width were 16 mm and 10 mm respectively. Similarly, the average tear length and width were 16 mm and 10 mm in the multiple anchors subgroup respectively. There was no significant difference in tear diameters (anteroposterior and mediolateral) between both subgroups (P = 0.99) (P = 0.64) respectively. Similarly, there was no significant difference in the operative time between both single anchor (14 minutes) and multiple anchors (17 minutes) subgroups (P = 0.19). For medium tears (mediolateral diameter 1–3 cm) with anteroposterior diameter ≤ 1 cm, in the single anchor subgroup, the average tear length and width were 10 mm and 16 mm respectively. Similarly, the average tear length and width were 10 mm and 16 mm in the multiple anchors subgroup respectively. There was no significant difference in tear diameters (anteroposterior and mediolateral) between both subgroups (P = 0.76) (P = 0.71) respectively. Similarly, there was no significant difference in the operative time between both single anchor (14 minutes) and multiple anchors (16 minutes) subgroups (P = 0.39). For medium tears (mediolateral diameter 1–3 cm) with anteroposterior diameter 1–3 cm, in the single anchor subgroup, the average tear length and width were 17 mm and 17 mm respectively. Similarly, the average tear length and width were 17 mm and 16 mm in the multiple anchors subgroup respectively. There was no significant difference in tear diameters (anteroposterior and mediolateral) between both subgroups (P = 0.44) (P = 0.07) respectively. Similarly, there was no significant difference in the operative time between both single anchor (16 minutes) and multiple anchors subgroups (19 minutes) (P = 0.40).

### Retear rate

**Fig 3:**
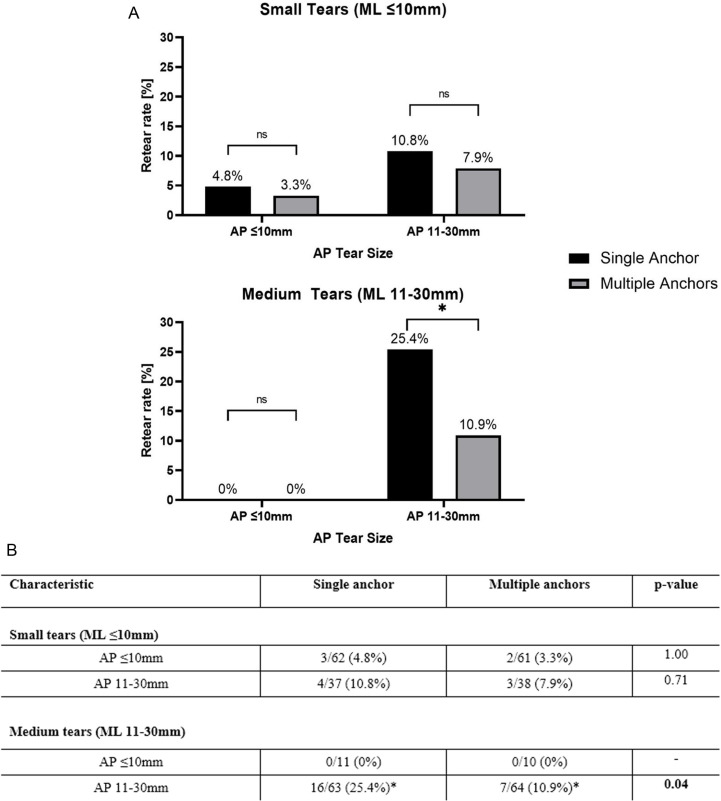
Clinical outcome at time zero, 6 weeks and 6 months postoperatively. Data is presented as mean with error bars derived from the standard error of mean. * Indicates a difference between groups with a P value <0.05.

[Fig 3] using ultrasound at 6 months postoperatively, For small tears (mediolateral diameter ≤ 1 cm) with anteroposterior diameter ≤ 1 cm, 3/62 patients (4.8%) had a retear in the single anchor subgroup and 2/61 patients (3.3%) had a retear in the multiple anchors subgroup. This difference was not statistically significant (P = 1.00). For small tears (mediolateral diameter ≤ 1 cm) with anteroposterior diameter 1–3 cm, 4/37 patients (10.8%) had a retear in the single anchor subgroup and 3/38 patients (7.9%) had a retear in the multiple anchors subgroup. This difference was not statistically significant (P = 0.71). For medium tears (mediolateral diameter 1–3 cm) with anteroposterior diameter ≤ 1 cm, there was no retears in single anchor and multiple anchors subgroups. For medium tears (mediolateral diameter 1–3 cm) with anteroposterior diameter 1–3 cm, 16/63 patients (25.4%) had a retear in the single anchor subgroup and 7/64 patients (10.9%) had a retear in the multiple anchors subgroup. This difference was statistically significant (P = 0.04).

### Clinical outcome

**Fig 4:**
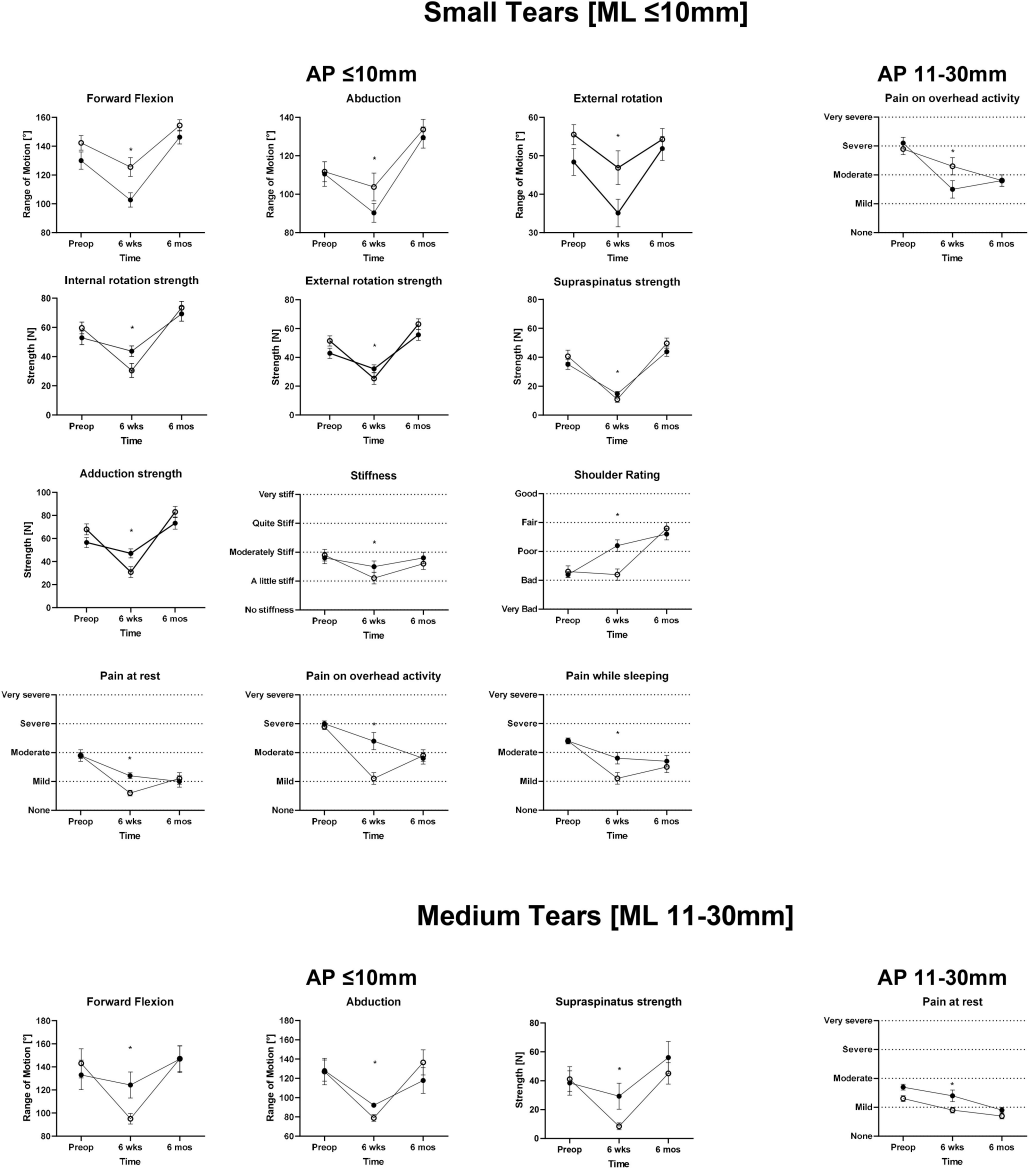
appendix - patient self-reported outcome questionnaire. Data is presented as mean with error bars derived from the standard error of mean. * Indicates a difference between groups with a P value <0.05.

Fig 4 Pre-operatively [Table 3]; the groups and subgroups patients had similar surgeon and patients reported outcomes. At 6 weeks post-operatively [Table 4]; For small tears (mediolateral diameter ≤ 1 cm) with anteroposterior diameter ≤ 1 cm, patients treated with a single anchor had significantly lower passive forward flexion (103 degrees), passive external rotation (35 degrees) and internal rotation strength (53 N) of their shoulders in comparison to patients with the same tear dimensions who were treated with multiple anchors (126 degrees) (47 degrees) (60 N) (P < 0.05). Additionally, the level of shoulder pain on overhead activity (2.4), at rest (1.2) and when sleeping (1.8) was significantly higher in patients treated with single anchor in comparison to patients treated with multiple anchors (0.6) (1.1) (1.1) (P < 0.05). However, patients in the single subgroup had significantly higher external rotation (32 N), supraspinatus (15 N) and adduction (47 N) strengths in comparison to patients in the multiple anchors subgroup (25 N) (11 N) (31 N) (P < 0.05). The overall satisfaction of the patients about their shoulders was significantly higher in patients treated with a single anchor (2.2) in comparison to patients treated with multiple anchors (1.2) (P < 0.05). For small tears (mediolateral diameter ≤ 1 cm) with anteroposterior diameter 1–3 cm, level of shoulder pain on overhead activity (1.5) was significantly lower in patients treated with single anchor in comparison to patients treated with multiple anchors (2.3) (P < 0.05). For medium tears (mediolateral diameter 1–3 cm) with anteroposterior diameter ≤ 1 cm, patients in the single anchor subgroup had significantly higher passive forward flexion (124 degrees), abduction (92 degrees) of their shoulders in comparison to patients treated with multiple anchors (95 degrees) (79 degrees) (P < 0.05). Additionally, patients in the single subgroup had significantly higher supraspinatus (29 N) strength in comparison to patients in the multiple anchors subgroup (8 N) (P < 0.05). For medium tears (mediolateral diameter 1–3 cm) with anteroposterior diameter 1–3 cm, level of shoulder pain at rest (1.4) was significantly higher in patients treated with a single anchor in comparison to patients treated with multiple anchors (0.9) (P < 0.05). At 6 months post-operatively [Table 5]; no significant difference was appreciated in either the patients or the physician reported outcomes between the groups or the subgroups.

**Table 3:** Pre-operative surgeon and patients reported outcome.

	ML ≤10mm				ML 11–30mm			
**Preoperative outcomes**	**AP ≤10mm**		**AP 11–30mm**		**AP ≤10mm**		**AP 11–30mm**	
	Single Anchor	Multiple Anchors	Single Anchor	Multiple Anchors	Single Anchor	Multiple Anchors	Single Anchor	Multiple Anchors
**Physician-rated outcomes**								
**Range of Motion [°] (higher is better)**								
Forward Flexion	130±6 (40–180)	142±5 (40–180)	129±6 (60–180)	146±5 (80–180)	133±13 (70–180)	143±13 (60–180)	138±5 (70–180)	142±5 (50–180)
Abduction	111±6 (30–180)	112±5 (30–180)	108±7 (30–180)	118±6 (60–180)	128±11 (80–170)	127±14 (60–180)	116±6 (40–180)	121±6 (40–180)
External rotation	48±4 (0–90)	56±3 (20–90)	48±4 (20–90)	48±4 (0–90)	66±9 (10–90)	47±6 (20–70)	50±3 (0–90)	51±3 (0–90)
**Strength [N] (higher is better)**								
Internal Rotation	53±5 (5–178)	60±4 (7–150)	49±5 (10–113)	51±5 (22–139)	60±9 (18–96)	49±8 (30–89)	58±4 (13–162)	59±4 (14–156)
External Rotation	43±4 (6–130)	51±4 (6–120)	40±4 (6–105)	41±4 (15–139)	51±9 (16–96)	46±8 (20–101)	50±4 (13–130)	49±4 (5–147)
Supraspinatus	35±4 (0–119)	41±4 (0–154)	32±5 (3–131)	33±4 (7–132)	38±8 (2–91)	41±9 (12–85)	42±5 (5–170)	41±4 (0–156)
Adduction	57±4 (6–170)	68±5 (5–171)	57±5 (12–140)	59±5 (24–159)	56±12 (3–135)	70±12 (28–145)	65±5 (12–232)	67±5 (2–177)
**Patient-reported outcomes**								
**Level of shoulder pain (higher is worse)**								
When resting	1.9±0.2	1.9±0.1	2±0.2	1.6±0.2	1.4±0.3	1.7±0.3	1.7±0.1	1.3±0.1
On overhead activity	3±0.1	2.9±0.1	3.1±0.2	2.9±0.2	2.9±0.4	2.6±0.4	3±0.1	2.8±0.1
When sleeping	2.4±0.1	2.4±0.1	2.6±0.2	2.2±0.2	2.3±0.3	2.2±0.3	2.5±0.1	2.3±0.1
**Shoulder stiffness (higher is worse)**								
	1.8±0.2	1.9±0.2	1.9±0.2	1.3±0.2	1.2±0.4	1.4±0.3	1.8±0.2	1.4±0.2
**Overall rating (lower is worse)**								
	1.2±0.1	1.3±0.2	1.1±0.2	1.3±0.2	1±0.3	0.8±0.3	1.3±0.1	1.3±0.1

P value < 0.05. All data expressed as mean ± SEM with range in bracket.

**Table 4:** Surgeon and patients reported outcome at 6 weeks post-operatively.

	ML ≤10mm				ML 11–30mm			
**6 weeks postop outcomes**	**AP ≤10mm**		**AP 11–30mm**		**AP ≤10mm**		**AP 11–30mm**	
	Single Anchor	Multiple Anchors	Single Anchor	Multiple Anchors	Single Anchor	Multiple Anchors	Single Anchor	Multiple Anchors
**Physician-rated outcomes**								
**Range of Motion [°] (higher is better)**								
Forward Flexion	103±5 (30–180)*	126±7 (70–180)*	118±7 (60–180)	106±6 (40–180)	124±11 (90–170)*	95±5 (80–120)*	105±5 (40–180)	101±4 (30–170)
Abduction	90±5 (30–180)	104±7 (30–180)	102±7 (40–180)	95±6 (30–180)	92±2 (85–100)*	79±4 (60–90)*	92±5 (30–180)	87±4 (30–170)
External rotation	35±4 (0–100)*	47±4 (10–90)*	39±4 (0–90)	37±5 (0–90)	46±7 (15–70)	36±10 (0–75)	36±4 (0–90)	28±3 (0–80)
**Strength [N] (higher is better)**								
Internal Rotation	53±5 (5–178)*	60±4 (7–150)*	49±5 (10–113)	51±5 (22–139)	60±9 (18–96)	49±8 (30–89)	58±4 (13–162)	59±4 (14–156)
External Rotation	32±3 (0–91)*	25±4 (0–96)*	28±3 (0–73)	29±2 (0–60)	36±7 (0–58)	28±6 (0–54)	28±2 (0–77)	33±2 (0–84)
Supraspinatus	15±2 (0–54)*	11±2 (0–61)*	12±2 (0–60)	16±2 (0–43)	29±9 (0–80)*	8±2 (0–19)*	13±2 (0–50)	15±2 (0–48)
Adduction	47±4 (0–130)*	31±5 (0–114)*	45±5 (0–173)	48±5 (0–118)	61±12 (0–118)	42±8 (0–69)	49±4 (0–183)	56±3 (0–134)
**Patient-reported outcomes**								
**Level of shoulder pain (higher is worse)**								
When resting	1.2±0.1*	0.6±0.1*	1±0.2	1.1±0.2	0.4±0.3	0.7±0.3	1.4±0.2*	0.9±0.1*
On overhead activity	2.4±0.3*	1.1±0.2*	1.5±0.3*	2.3±0.3*	1.7±0.8	2.3±0.8	2.3±0.2	2.2±0.2
When sleeping	1.8±0.2*	1.1±0.2*	1.6±0.2	1.5±0.2	1.1±0.3	1.7±0.6	1.9±0.2	1.5±0.1
**Shoulder stiffness (higher is worse)**								
	1.5±0.2	1.1±0.2	1.2±0.2	1.6±0.2	0.6±0.3	1.5±0.5	1.6±0.2	1.6±0.2
**Overall rating (lower is worse)**								
	2.2±0.2*	1.2±0.2*	2.6±0.2	2.3±0.2	2.6±0.6	1.4±0.5	2.4±0.2	2.6±0.1

*Represents p<0.05. All data expressed as mean ± SEM with range in brackets.

**Table 5:** Surgeon and patients reported outcome at 6 months post-operatively.

	ML ≤10mm				ML 11–30mm			
**6 months postop outcomes**	**AP ≤10mm**		**AP 11–30mm**		**AP ≤10mm**		**AP 11–30mm**	
	Single Anchor	Multiple Anchors	Single Anchor	Multiple Anchors	Single Anchor	Multiple Anchors	Single Anchor	Multiple Anchors
**Physician-rated outcomes**								
**Range of Motion [°] (higher is better)**								
Forward Flexion	146±5 (30–180)	154±4 (80–180)	143±7 (40–180)	144±6 (30–180)	147±12 (90–180)	147±11 (90–180)	138±5 (50–180)	148±5 (80–180)
Abduction	130±6 (30–180)	134±5 (69–180)	124±7 (40–180)	131±8 (30–180)	118±13 (80–180)	137±13 (90–180)	128±5 (50–180)	131±6 (50–180)
External rotation	52±3 (0–90)	54±3 (0–90)	55±4 (0–90)	55±3 (20–90)	59±7 (20–80)	40±6 (20–70)	48±3 (10–90)	51±3 (0–100)
**Strength [N] (higher is better)**								
Internal Rotation	69±5 (3–172)	74±4 (26–155)	58±4 (11–101)	53±5 (0–117)	65±10 (10–99)	59±8 (22–96)	64±4 (14–148)	63±4 (15–120)
External Rotation	56±4 (6–144)	63±4 (18–130)	48±3 (13–97)	45±4 (0–121)	57±9 (6–84)	50±7 (10–86)	50±4 (12–131)	52±3 (14–121)
Supraspinatus	44±3 (0–110)	50±4 (10–120)	44±4 (5–104)	34±4 (0–97)	56±11 (5–97)	45±7 (20–102)	41±4 (0–120)	44±4 (4–104)
Adduction	73±5 (3–181)	83±5 (12–185)	62±5 (7–149)	66±6 (0–162)	77±11 (15–114)	67±12 (24–140)	68±4 (12–133)	71±4 (15–138)
**Patient-reported outcomes**								
**Level of shoulder pain (higher is worse)**								
When resting	1±0.2	1.1±0.2	0.9±0.2	0.7±0.2	0.3±0.2	0.8±0.4	0.9±0.1	0.7±0.1
On overhead activity	1.8±0.2	1.9±0.2	1.8±0.2	1.8±0.2	1.4±0.6	1.4±0.5	1.9±0.2	1.6±0.2
When sleeping	1.7±0.2	1.5±0.2	1.3±0.2	1.1±0.2	1.2±0.5	1.4±0.5	1.4±0.2	1.3±0.2
**Shoulder stiffness (higher is worse)**								
	1.8±0.2	1.6±0.2	1.4±0.2	1.2±0.2	1.3±0.4	1.2±0.4	1.4±0.2	1.1±0.2
**Overall rating (lower is worse)**								
	2.6±0.2	2.8±0.2	2.8±0.2	3.1±0.2	3.4±0.2	2.6±0.4	2.9±0.1	3±0.1

P value < 0.05. All data expressed as mean ± SEM with range in brackets.

## Discussion

Different studies evaluated the biomechanical and the clinical efficacy of single-row technique for rotator cuff tears repair. Our surgeon preferred using the single row technique all through his practice as different studies didn’t show an inclusive advantage for one repair technique over another. For Example, Biomechanically, Nelson CO et al. [34] found that single row repair technique had similar biomechanical strength after cyclic loading as double-row repair technique.

Clinically, Aydin N et al. [35] concluded that single-row repair was an effective technique compared to double-row and trans osseous equivalent techniques for repair of rotator cuff tears with mediolateral diameter ≤ 3 cm. Ming Chen et al. [20] suggested that rotator cuff tears with a mediolateral diameter ≤ 3 cm which have been repaired using single-row repair technique had similar structure integrity as those repaired using the double-row technique. Grasso A et al. [17] followed 80 patients with full-thickness rotator cuff tears who underwent arthroscopic repair with suture anchors in either single-row technique (40 patients) or double-row technique (40 patients). They found that patients who had their cuff repaired using the single-row technique had similar clinical outcome after 2 years compared to patients who had their cuff repaired using double-row repair technique. However, to our knowledge, there are no studies comparing the rotator cuff integrity and the clinical outcome after using one in comparison to multiple anchors for repair of rotator cuff tears.

Therefore, the aim of this study was to determine if the usage of a single anchor in single row rotator cuff repair would give a similar retear rate and clinical outcome in comparison to the usage of multiple anchors in rotator cuff tears repair in a matched group of patients after 6 months of repair. We hypothesized that using a single anchor for cuff tears sized ≤ 3*3 cm² in a single row repair technique would result in a similar retear rate and clinical outcome after 6 months of repair compared to the usage of multiple anchors in similar sized tears. Interestingly, there was no significant difference in rotator cuff retears or clinical outcome between patients with cuff tears ≤ 3*3 cm² who had their tears repaired using a single anchor in comparison to those who had their tears repaired using multiple anchors in a single-row technique unless the anteroposterior and mediolateral diameters of the tear were both larger than 1 cm, for which the utilization of multiple anchors showed an advantage over using a single anchor for repair with significantly lower retears at 6 months post-surgery. Additionally, it is worth mentioning that the operative time was significantly shorter only when a single anchor was used for repair of tears ≤ 1 cm in both dimensions. This finding may suggest that the cost of surgery can be reduced if the proper amount of anchors were used for repair. However, further investigation is needed to assert this claim.

With the exception of medium tears with an anteroposterior diameter > 1 cm that mandates the utilization of multiple anchors for repair and coverage of the large footprint of the tear, a proposed explanation behind the similarity in the structural integrity and the clinical outcome between patients with tears repaired by a single or multiple anchors after 6 months of repair could be the unequal distribution of the pulling force applied to the anchors used; making one of the anchors as the main one receiving the highest amount of tension even when multiple anchors were used, this would transform the multiple anchors model again into a single anchor one, yielding to a comparable outcome to those who were treated with a single anchor from the beginning. Therefore, further biomechanical and clinical studies may provide a better understanding and a solid recommendation for the optimal position and the exact number of anchors needed to equalize the distribution of the pulling forces throughout the model used for repair which may improve the structural integrity of the repaired rotator cuffs and the clinical outcome post repair.

### Limitations

All patients in this study were enrolled and matched without blinding or randomization. In addition, the follow up period for the patients post repair was only for 6 months; therefore, a long-term clinical outcome could not be assessed. Furthermore, this study did not involve tears larger than 3*3 cm² in either anteroposterior or mediolateral dimensions, as those tears inherently require more than a single anchor for their repair and for coverage of the footprint.

## Conclusion

6 months postoperatively; there was no significant difference in the retear rate or clinical outcome between patients with small and medium cuff tears (≤ 3 * 3 cm²) who had their cuff tears repaired using a single anchor in comparison to those who had their cuff tears repaired using multiple anchors unless both the mediolateral and anteroposterior diameters of the tear were larger than 1 cm, for which the utilization of multiple anchors showed an advantage over using a single anchor for repair with significantly lower retears at 6 months post-surgery. Operative time was significantly shorter only when a single anchor was used for repair of tears ≤ 1 cm in both mediolateral and anteroposterior diameters.

## Supporting information

S1 File Anonymized_Anchor_Data. (CSV)
