# Correction: Is it enough to utilize a single anchor for repair of rotator cuff tears ≤ 3 * 3 cm²?

**DOI:** 10.1371/journal.pone.0340485

**Published:** 2026-01-05

**Authors:** Ala’ Hawa, Alexander Tham, James Bilbrough, Christyon Hayek, Mina Shenouda, George A. C. Murrell

The captions for Figs 1–4 are incorrect. The captions have been provided here:

**Fig 1:**
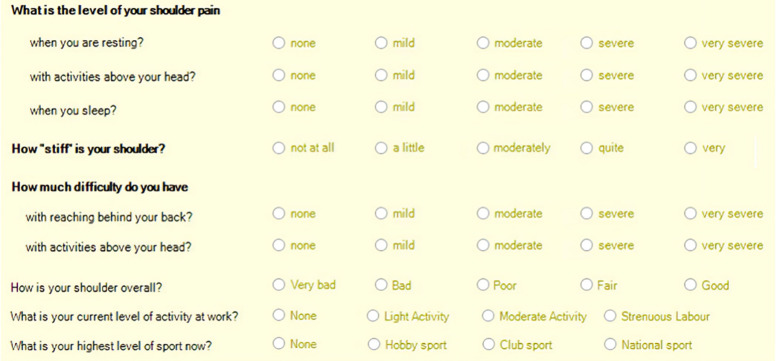
Appendix. Patient self-reported outcome questionnaire. Data is presented as mean with error bars derived from the standard error of mean. * Indicates a difference between groups with a P value <0.05.

**Fig 2:**
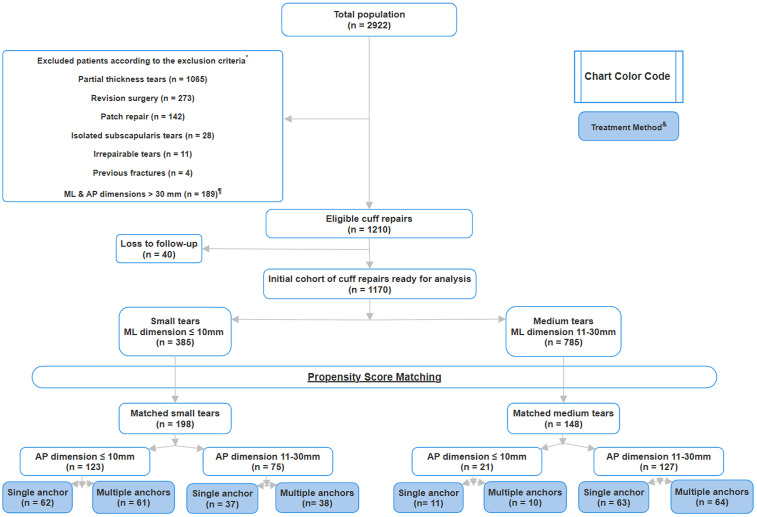
Patients exclusion hierarchy. * Represents a list for patients excluded according to our exclusion criteria. ¶ ML is the mediolateral dimension of the tear, AP is the anteroposterior dimension of the tear & the “faint blue” color coded boxes represent the treatment method used for repair of rotator cuff tears.

**Fig 3:**
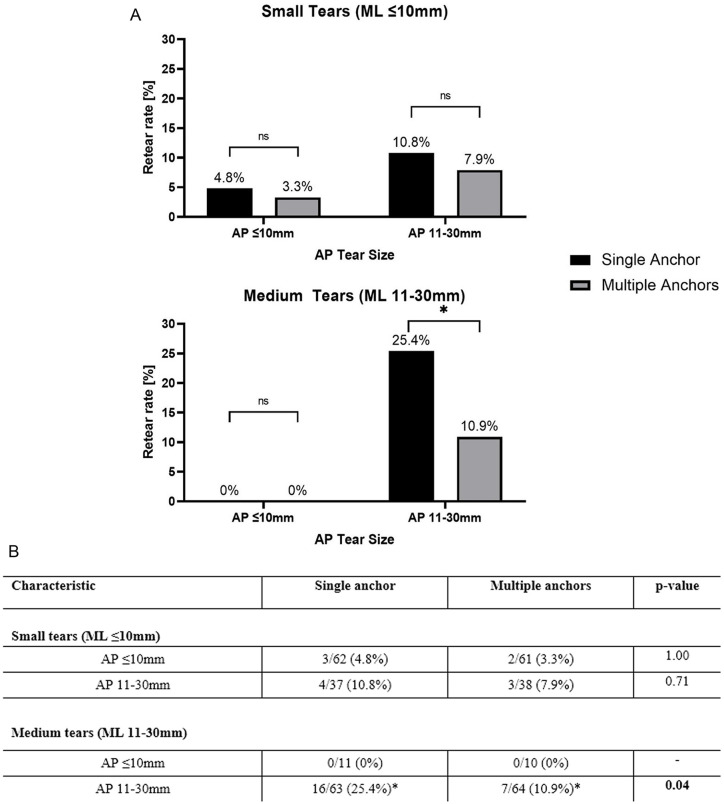
(A & B) Graphical & Tabular representations of retear rates 6 months postoperatively for both single & multiple anchors groups among tear areas of size ML. ≤ 10 mm * AP ≤ 10 mm, ML ≤ 10 mm * AP 11-30 mm, ML 11-30 mm * AP ≤ 10 mm and ML 11-30 mm * AP 11-30 mm. Black bars represent the Single Anchor group, and grey bars represent Multiple Anchors.

**Fig 4:**
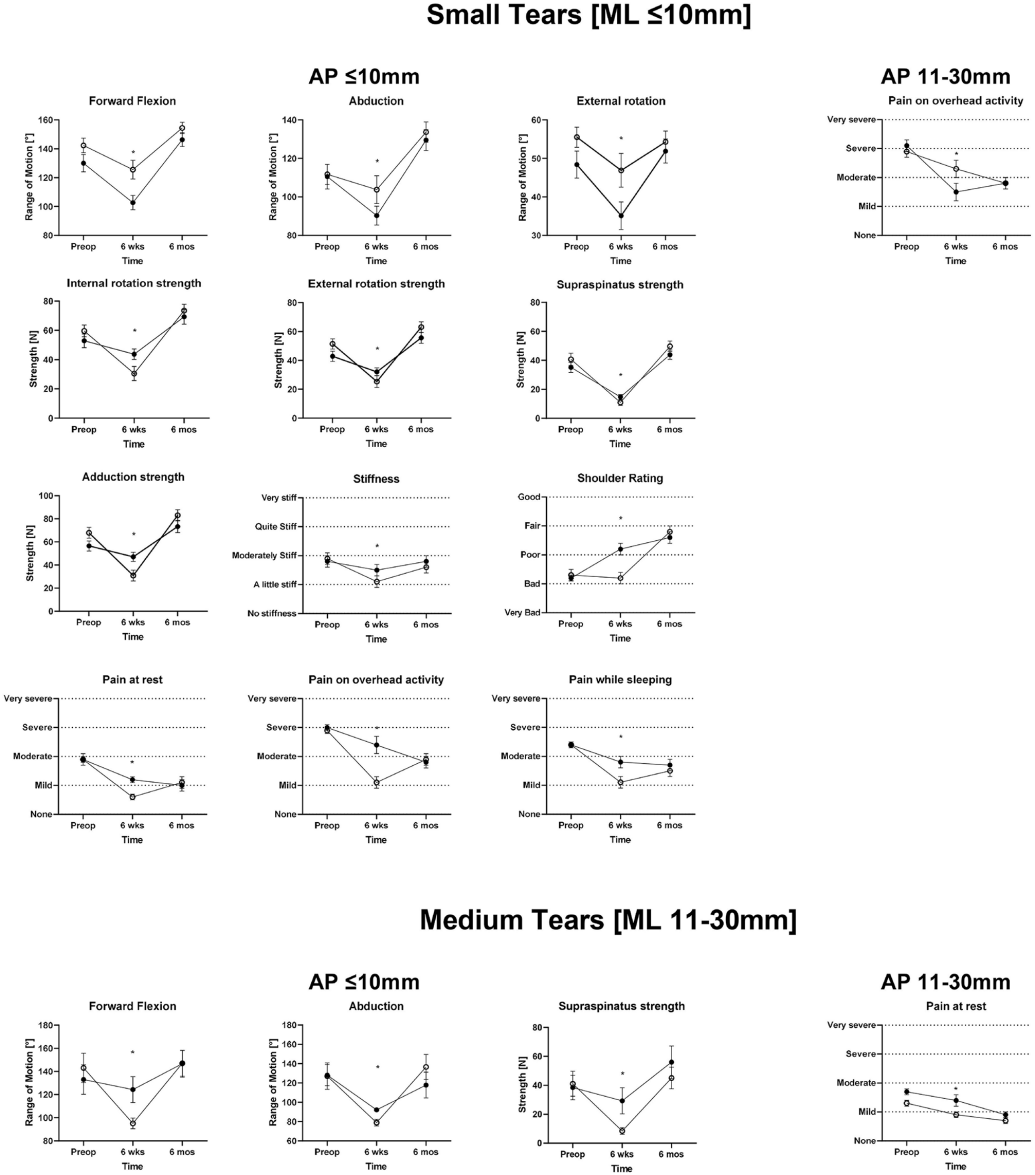
Clinical outcome at time zero, 6 weeks and 6 months postoperatively. Data is presented as mean with error bars derived from the standard error of mean. * Indicates a difference between groups with a P value <0.05.
